# Role of NLRP3 Inflammasome in the Progression of NAFLD to NASH

**DOI:** 10.1155/2016/6489012

**Published:** 2016-05-04

**Authors:** Xingyong Wan, Chengfu Xu, Chaohui Yu, Youming Li

**Affiliations:** Department of Gastroenterology, The First Affiliated Hospital, College of Medicine, Zhejiang University, Hangzhou, Zhejiang 310003, China

## Abstract

Nonalcoholic fatty liver disease (NAFLD) has been recognized as a major public health problem worldwide. Nonalcoholic steatohepatitis (NASH) is an advanced form of NAFLD that may progress to cirrhosis and hepatocellular carcinoma. The pathogenesis of disease progression from NAFLD to NASH has not been fully understood. Immunological mechanisms that have been increasingly recognized in the disease progression include defects in innate immunity, adaptive immunity, Toll-like receptor (TLR) signaling, and gut-liver axis. The NLRP3 inflammasome is an intracellular multiprotein complex involved in the production of mature interleukin 1-beta (IL-1*β*) and induces metabolic inflammation. NLRP3 inflammasome has been recently demonstrated to play a crucial role in the progression of NASH. This review highlights the recent findings linking NLRP3 inflammasome to the progression of NASH.

## 1. Introduction

Nonalcoholic fatty liver disease (NAFLD) is one of the most common chronic liver diseases worldwide [1]. It affects more than one-third of adults in Western countries [2]. The spectrum of NAFLD includes simple steatosis, steatohepatitis (NASH) with or without fibrosis, cirrhosis, and hepatocellular carcinoma [2]. Around 10–20% patients with NAFLD will eventually develop NASH [3], which has been predicted to be the leading indication for liver transplantation in the USA by the year 2020 [4]. The current mechanism is thought to be based on a “two-hit” hypothesis, which was proposed in 1998 [5]. The first hit, which includes simple steatosis and insulin resistance, sensitizes the liver for later events leading to the development of steatohepatitis [6]. The second hit is a multifactorial process including inflammation, oxidative stress, lipid peroxidation, and mitochondrial dysfunction [7]. All of these insults contribute to the progression of NAFLD to NASH and cirrhosis. However, the detailed mechanism of the progression from NAFLD to NASH has not been completely explained so far, and, therefore, no effective treatment has been developed so far.

The NLRP3 inflammasome is a large intracellular multiprotein complex, which consists of an inflammasome sensor molecule (typically a NOD-like receptor [NLR]), and adaptor proteins, such as apoptosis-associated speck-like protein containing a caspase-recruitment domain (ASC) and the precursor procaspase-1 [8]. The inflammasome can recognize a range of substances including pathogen associated molecular patterns (PAMPs), danger associated molecular patterns (DAMPs), and environmental irritants [8, 9]. There are two steps involved in the activation of NLRP3 inflammasome. The first step is Toll-like receptor- (TLR-) mediated upregulation of NLRP3 inflammasome components and the procytokines, prointerleukin-1 beta (IL-1*β*), and pro-IL-18 [8, 10]. The second step is the assembly of NLRP3 inflammasome complex, including the NOD-like receptor NLRP3, the cytosolic protein ASC, and procaspase-1 [8, 10]. A variety of activators can provide step 2, including crystals, large particles such as silica, asbestos, urate, and ATP via the P2X7 receptor, and reactive oxygen species [8, 10]. NLRP3 inflammasome activation leads to the maturation of caspase-1, which further cleaves pro-IL-1*β* and pro-IL-18 into mature forms and results in their secretion from the cell. The activation of caspase-1 also induces the release of IL-1*α* [9].

Recently, the expression of NLRP3 and its components was observed to be significantly increased both in murine models and in humans with NASH [11–13]. Moreover, using gene-knockout mice or pharmacological inhibition of these genes resulted in the alleviation of hepatic steatosis, hepatocyte inflammation, and fibrogenesis [13–24]. These results suggest that NLRP3 inflammasome may play a critical role in the development of NASH and may act as a molecular therapeutic target. This review highlights the current knowledge of NLRP3 inflammasome in relation to NASH.

## 2. Pathogenesis of NASH

The current most persuasive mechanism of the disease progression in NASH is lipotoxicity [25–27]. Lipotoxic injury appears to occur due to excessive flux of free fatty acids (FFAs) through hepatocytes [25, 26]. Free fatty acids (FFAs), especially saturated fatty acids (SFAs), are derived from diet, adipose tissue lipolysis, and* do novo* lipogenesis from glucose [25]. Under physiological conditions, SFAs can be transferred to mitochondria for *β*-oxidation, or secreted into plasma as esterified very low density lipoproteins, or stored as lipid droplets [25, 26]. The excessive SFAs can generate lipotoxic intermediates, such as phosphatidic acid, lysophosphatidic acid, lysophophatidylcholine, ceramides, and diacylglycerols [25]. Lipotoxic intermediates can induce endoplasmic reticulum (ER) stress with accumulation of unfolded or misfolded proteins in ER, mitochondrial dysfunction, reactive oxygen species (ROS) formation, and oxidative stress, all of which lead to apoptosis, a key pathogenic feature of NASH [26]. Binding of SFAs with TLR4 can lead to a series of cascade reactions, including proinflammatory nuclear factor-kappa B (NF-*κ*B) pathway activation, proapoptotic apoptosis signal-regulating kinase 1/c-Jun N-terminal kinase (ASK1/JNK) pathway activation, and mitochondrial dysfunction amplification [26]. Insulin resistance plays a central role in adipose tissue lipolysis and peripheral glucose disposal, which causes an excessive FFA traffic and is critical to the development of oxidative stress and lipotoxicity [25, 27]. Increasing attention is being given to dietary factors as a cause of NASH progression. Recently, polyunsaturated fatty acids (PUFAs), fructose, and high dietary cholesterol have been shown to play an important role in the NAFLD/NASH development. PUFAs consist of omega- (*ω*-) 3 PUFAs and *ω*-6 PUFAs; the former reduces while the latter promote inflammation [28]. Lipidomic analysis of NAFLD and NASH patients showed upregulation of *ω*-6/*ω*-3 ratio in liver tissues [29, 30]. The presence of *ω*-6 PUFAs in plasma can help to differentiate patients with NAFLD from those with NASH [31]. Animal studies using high-fat diet (HFD) with high dietary *ω*-6/*ω*-3 ratio developed more severe steatohepatitis when compared with animals that were fed a HFD with low dietary *ω*-6/*ω*-3 ratio [32]. Fructose consumption can increase fat mass,* de novo* lipogenesis, and inflammation and induce insulin resistance [33]. Several studies have indicated that the development of NAFLD may be associated with excessive dietary fructose consumption [33–35]. Further, a cross-sectional analysis involving 427 adult liver biopsies confirmed the different stages of NAFLD adults from NASH Clinical Research Network demonstrating that daily fructose ingestion was associated with increased fibrosis, after controlling for age, gender, body mass index, and total caloric intake [33]. High dietary cholesterol is an activator of liver X receptor, which alters the balance between storage and oxidation of fatty acids, leading to excessive FFA flux, which drives lipotoxic injury of hepatocytes [36]. A large epidemiological survey conducted in the US reported that dietary cholesterol consumption was independently associated with the development of cirrhosis [37]. Animal studies also demonstrated high dietary cholesterol to be a critical factor in the development of NASH [36]. Pharmacological resolution of cholesterol crystals has been found to ameliorate fibrotic NASH in high-fat high-cholesterol induced NASH model [38]. Another study has indicated that the presence or distribution of hepatic cholesterol crystals can distinguish NASH from simple steatosis in humans and mice [39]. Recently, the development of NASH has been linked to a heritable disease. Human genome-wide association studies (GWAS) identified the genetic variant of patatin-like phospholipase domain containing three genes, that is,* PNPLA3*,* rs738409*, and* I148M* as a strong predictor of NASH and simple steatosis [40, 41].

## 3. Immunological Mechanisms Involved in the Pathogenesis of NASH

Both innate and adaptive immune mechanisms play important roles in the development of NASH. The innate immune cells in the liver are comprised of large numbers of Kupffer cells (KCs), natural killer T (NKT) cells, and others. KCs contribute to the liver injury mainly through proinflammatory cytokines release, chemokine induction, and monocyte recruitment [42]. The depletion of KCs has been found to attenuate methionine- and choline-deficient diet (MCD) and HFD-induced liver injury, steatosis, and proinflammatory monocyte infiltration [42]. The NKT cells regulate both the immune responses by secreting proinflammatory/antifibrotic Th1 cytokines and anti-inflammatory/profibrotic Th2 cytokines after stimulation [43]. Further, these cells may play an important role in the progression of inflammation and fibrosis in NASH. Animal studies indicated that NKT cell-deficient mice had dramatically less fibrosis in MCD-induced NASH model, which may have been due to inactivation of hedgehog pathway and decreased osteopontin expression [43, 44]. The adaptive immune cells include T lymphocytes and B lymphocytes. In NASH patients and animal models, CD4(+) and CD8(+) T cell infiltration was increased [45, 46]. In human liver biopsies taken from NAFLD/NASH patients, CD4(+) and CD8(+) T cell infiltration was positively correlated with NASH progression [45]. CD4(+) T cells may promote hepatic inflammation through upregulation of interferon-gamma (IFN-*γ*) and CD40 ligand, both of which have been shown to be associated with inflammation in liver [46]. The role of CD8(+) T cells in NASH progression is not clearly understood and requires further research. Serum levels of B-cell-activating factor (BAFF) were increased in NASH patients compared with patients with simple steatosis [47]. Animal studies using BAFF−/− mice showed improvement in HFD-induced obesity and insulin resistance, the two well-recognized risk factors of NASH progression [47].

Toll-like receptors are members of pattern-recognition receptor superfamily that play important role in the activation of innate immune system. These receptors can recognize a wide variety of PAMPs and some types of DAMPs derived from dying host cells [48]. To date, 10 and 13 TLRs have been identified in humans and mice, respectively [49]. Further, TLR2, TLR4, and TLR9 have been reported to be associated with NAFLD/NASH [50]. The role of TLR2 in the pathogenesis of NAFLD is controversial. In HFD-induced mice, TLR2 deficiency reduces hepatic steatosis [51]. Likewise, in choline-deficient amino-acid- (CDAA-) defined diet-induced NASH models, TLR2 and palmitic acid cooperatively activate the inflammasome in KCs, and TLR2 deficiency showed diminished inflammasome activation and decreased liver inflammation and fibrosis. However, hepatic steatosis has been reported to be independent of TLR2 signaling [50]. On the contrary, two studies have reported that the deficiency of TLR2 results in increased liver injury in MCD-induced NASH model [52, 53]. Several reports have demonstrated the importance of TLR4 signaling in the development of NASH. The role of TLR4 has been shown in HFD, MCD, high-fat and high-cholesterol (HFHC), high-fat and high-fructose diets models of NAFLD/NASH. In HFD-induced NAFLD model, TLR-4/MyD88 signaling in liver parenchymal cells plays a pivotal role during the early progression of NAFLD, wherein high-mobility group protein B1 (HMGB1) served as a TLR4 activator [54]. Mice deficient in TLR-4 showed significantly lower liver injury and lipid accumulation in mice fed on MCD diet [55]. TLR-4 in KCs plays a key role in HFHC diet-induced NASH, partly via inducing ROS-dependent activation of X-box binding protein-1 (XBP-1) [56]. C3H/HeJ TLR4-mutant mice showed reduced lipid accumulation, hepatocellular ballooning, infiltration, liver injury, and fibrogenesis in different stages of NAFLD development compared with C3H/HeN wild-type mice fed on a high-fat and high-fructose diet [57]. Moreover, TLR4 codon 299 heterozygous gene mutation (Asp299Gly) in humans may have a preventive role against the development of NAFLD [58]. In CDAA diet-induced NASH, TLR9-deficient mice showed reduced hepatic steatosis, inflammation, and fibrosis, and these differences mainly depended on IL-1*β* produced by KCs [17]. In MCD-induced NASH, NLRP3 inflammasome sensors and inflammasome activation involved both BM-derived and non-BM-derived cells in the liver via HMBG1-TLR9-MyD88 pathways [59].

There is increasing evidence, which shows that gut microbiota, gut-derived endotoxin, and intestinal hyperpermeability play an important role in the pathogenesis of NAFLD/NASH [60, 61]. Gut microbiota are a source of bacterial products such as lipopolysaccharide (LPS), bacterial DNA, and peptidoglycan, all of which are delivered to the liver through the portal vein [62]. Increased serum LPS levels and gut permeability were associated with NAFLD development, and serum LPS levels are increased in NASH patients [63]. Administration of probiotics results in decreased hepatic steatosis, inflammation, liver injury, and fibrosis in different NAFLD animal models [64, 65]. Microbial danger signals which are absorbed due to increased gut permeability are recognized by pattern-recognition receptors including TLRs, such as TLR2, TLR4, and TLR9 [60]. Based on the above reports we can hypothesize that NLRP3 inflammasome-driven microflora may play a key role in NAFLD development.

## 4. The Role of NLRP3 Inflammasome in NASH Pathogenesis

The presence of NLRP3 inflammasome and/or NLRP3 inflammasome activation has been shown in innate immune cells, including KCs, neutrophils, and dendritic cells, as well as nonimmune cells, including hepatocytes, hepatic stellate cells, endothelial cells, and myofibroblasts [8, 49]. NLRP3 inflammasome activation in different cell types has not been completely elucidated. One group showed that KCs, the largest population of liver macrophages, produced large quantities of IL-1*β* [17]. Another group identified KCs as a key cellular source of active caspase-1 in MCD-induced NASH [21]. Lack of IL-1*α* production by liver parenchymal cells, rather than bone-marrow-derived cells, was found to protect against the development of steatohepatitis and fibrosis [18]. Selective deficiency of IL-1*α* in KCs reduced liver inflammation and expression of inflammatory cytokines, which may block the progression of steatosis to steatohepatitis [66]. NLRP3 inflammasome activation may also involve the coordinated interplay between hepatocytes and KCs, as demonstrated by experiments in which palmitic acid-induced apoptosis caused the release of danger signals form hepatocytes, which then activated the inflammasome in liver mononuclear cells [11]. To further dissect the contribution of NLRP3 inflammasome in different cell types, experiments involving conditional gene-knockout mice will have to be developed [67].

The NLRP3 inflammasome activation has been mainly associated with NASH, but not with steatosis. Several studies have shown that the gene expression of NLRP3 inflammasome components, pro-IL-18, and pro-IL-1*β* was markedly increased in the liver of NASH patients [11, 13]. The mRNA levels of pro-IL-1*β* were significantly correlated with levels of* COL1A1*, a key profibrogenic gene [13]. The mRNA levels of NLRP3 inflammasome components, caspase-1 activity, and serum IL-1*β* were significantly increased in MCD and long-term HFD-induced mice model of NASH, but not in short-term HFD fed or leptin-deficient (ob/ob) mice [11]. In atherogenic HFD-induced NASH model, the mRNA levels of NLRP3, ASC, pro-IL-1*β*, and procaspase-1 were significantly increased [12]. In hepatocytes of MCD diet-fed mice, increased expression of NLRP3, ASC, procaspase-1, pro-IL-1*β* mRNA was found [11]. Palmitic acid (a saturated fatty acid), but not unsaturated fatty acid, can induce NLRP3 inflammasome activation and IL-*β* release via TLR4, in the presence of LPS [11, 68]. Furthermore, palmitic acid-induced inflammasome activation was dependent on adenosine monophosphate-activated protein kinase- (AMPK-) autophagy-ROS signaling axis, and this pathway is considered to be a specialized route of inflammasome activation only in response to palmitic acid [68].

## 5. The Role of NLRP3 Inflammasome in NASH Intervention Treatment

To date, no therapeutics directly targeting the NLRP3 inflammasome have been developed in clinical research. However, novel tools employing gene-knockout mice have provided positive evidence in animal models of NASH. After 16 weeks of HFD feeding, ASC-deficient mice were protected from HFD-induced liver steatosis, but there was no effect in NLRP3 and caspase-1-deficient mice [19]. The histological evidence suggested that NLRP3-deficient mice had reduced hepatic steatosis compared with wild-type mice fed on HFD [20]. On the contrary, another study showed that the knockout of NLRP3 inflammasome components and associated proteins increased hepatic steatosis, inflammation, and liver injury in mice fed on MCD diet [69]. A recent study demonstrated that NLRP3 knockout mice fed with a 16 weeks of CDAA diet were protected from hepatomegaly, liver injury, hepatocyte inflammation, and liver fibrosis [13]. Moreover, in a 4-week CDAA fed mice, NLRP3 knock-in resulted in chemokine production, liver inflammatory changes, hepatic stellate cell (HSC) activation, and liver fibrogenesis [13]. Caspase inhibition as a novel therapeutic target for treatment of NAFLD/NASH is currently attracting attention. In MCD diet-fed db/db mice, prolonged treatment with pan-caspase inhibitor, VX-166, in conjunction with the diet, showed reduced hepatic triglycerides at 4 weeks and improved hepatic fibrogenesis. However, no effect on diet-induced liver injury, including hepatic inflammation and ballooning, overall NAFLD activity score, and ALT levels was observed [15]. Another study reported that, in both models (HFD and MCD), VX-166 did not reduce steatosis but reduced hepatic apoptosis, histological inflammation, serum ALT levels, and oxidative stress [16]. In the HFD-induced NASH mice model, pan-caspase inhibitor, emricasan reduced hepatic apoptosis, liver injury, and hepatic inflammation and markedly reduced liver fibrogenesis but did not lead to reduction in hepatic steatosis [24]. Selective inhibition of caspase-1 in HFD or MCD diet-induced NASH protects against liver injury, hepatic inflammation, and fibrosis [21, 23]. A phase 2, randomized, double-blind, placebo-controlled trial showed that 4 weeks' treatment with GS-9450, a selective caspase inhibitor (caspases 1, 8, and 9), resulted in significant and dose-dependent reductions in alanine transaminase (ALT) levels and smaller nonstatistically significant reductions in aspartate transaminase (AST) and cytokeratin-18 (CK-18) fragments in patients with NASH [70]. Further animal and human studies are required to elucidate the therapeutic role of selective caspase-1 inhibitors in NASH.

Interleukin-1 signaling is also thought to play a key role in NAFLD/NASH development. IL-1*β* promoted lipid accumulation, hepatocyte injury, and HSC activation in cultured hepatocytes or HSCs from WT and TLR9−/−, but not in IL-1R−/− mice fed with a CDAA diet [17]. IL-1*α* and IL-1*β* knockout mice had reduced liver damage, decreased progression of steatosis to NASH, and reduced liver fibrosis in atherogenic diet-induced NASH [18]. IL-1 receptor (IL-1R) knockout mice exhibit reduced steatosis, hepatocyte damage, and fibrosis [17]. IL-1 receptor antagonist (IL-1RA) knockout mice had exacerbated hepatic fat accumulation, liver damage, and fibrosis when fed with an atherogenic diet [14]. In another study, serum IL-1RA levels were associated with NASH in obese individuals, the degree of lobular inflammation in liver and serum ALT in a population study involving 6447 men, which was independent of body mass index (BMI), alcohol consumption, and insulin sensitivity [22].

Several mutually contradictory results have been reported in the literature on the issue. One reason for this could be the nonavailability of murine models exhibiting the entire phenotypic spectrum of clinical NAFLD/NASH. The MCD diet induces the phenotypic changes of NASH in the liver. However, it does not produce characteristic features found in NASH patients, such as obesity and insulin resistance. HFD can induce obesity, insulin resistance, and a chronic state of low-grade inflammation; however the pathological change found in liver is predominantly steatosis.

## 6. Conclusion

NAFLD has become a serious public health problem in recent years. The mechanisms of the progression from NAFLD to NASH remain poorly understood. Immunological mechanisms, including defects in innate immunity, adaptive immunity, TLR receptor signaling, and gut-liver axis, have been recognized to play an important role in the disease progression. NLRP3 inflammasome activation can lead to NAFLD development and progression, including hepatic steatosis, inflammation, liver injury, and fibrogenesis. NLRP3 inflammasome may serve as a novel therapeutic target for the treatment of NAFLD and NASH.
